# Risk factors associated with *Pneumocystis jirovecii* pneumonia in non-HIV immunocompromised patients and co-pathogens analysis by metagenomic next-generation sequencing

**DOI:** 10.1186/s12890-022-02300-8

**Published:** 2023-02-24

**Authors:** Liping Huang, Shuyun Xu, Zhimin Huang, Yusheng Chen, Nengluan Xu, Baosong Xie

**Affiliations:** 1 Shengli Clinical Medical college of Fujian Medical University, Fuzhou, 350001 Fujian Province China; 2 Department of Respiratory and Critical Care Medicine, Fujian Provincial Hospital, Fuzhou, 350001 Fujian Province China

**Keywords:** *Pneumocystis jirovecii* pneumonia (PJP), Immunocompromised patients, Metagenomic next-generation sequencing (mNGS), Risk factors, Bronchoalveolar lavage fluid (BALF)

## Abstract

**Background:**

*Pneumocystis jirovecii* pneumonia (PJP) is one of the most common opportunistic infections in immunocompromised patients. However, the accurate prediction of the development of PJP in non-HIV immunocompromised patients is still unclear.

**Methods:**

Non-HIV immunocompromised patients confirmed diagnosis of PJP by the clinical symptoms, chest computed tomography and etiological results of metagenomic next-generation sequencing (mNGS) were enrolled as observation group. Another group of matched non-HIV immunocompromised patients with non-PJP pneumonia were enrolled to control group. The risk factors for the development of PJP and the co-pathogens in the bronchoalveolar lavage fluid (BALF) detected by mNGS were analyzed.

**Results:**

A total of 67 (33 PJP, 34 non-PJP) participants were enrolled from Fujian Provincial Hospital. The ages, males and underlying illnesses were not significantly different between the two groups. Compared to non-PJP patients, PJP patients were more tends to have the symptoms of fever and dyspnea. The LYM and ALB were significantly lower in PJP patients than in non-PJP patients. Conversely, LDH and serum BDG in PJP patients were significantly higher than in non-PJP controls. For immunological indicators, the levels of immunoglobulin A, G, M and complement C3, C4, the numbers of T, B, and NK cells, had no statistical difference between these two groups. Logistic multivariate analysis showed that concomitant use of corticosteroids and immunosuppressant (*OR* 14.146, *P* = 0.004) and the lymphocyte counts < 0.7 × 10^9^/L (*OR 6.882*, *P* = 0.011) were risk factors for the development of PJP in non-HIV immunocompromised patients. 81.82% (27/33) and 64.71% (22/34) mixed infections were identified by mNGS in the PJP group and non-PJP group separately. *CMV*, *EBV* and *Candida* were the leading co-pathogens in PJP patients. The percentages of *CMV* and *EBV* identified by mNGS in PJP group were significantly higher than those in the control group(*p* < 0.005).

**Conclusions:**

Clinicians should pay close attention to the development of PJP in non-HIV immunocompromised patients who possess the risk factors of concomitant use of corticosteroids and immunosuppressant and the lymphocyte counts < 0.7 × 10^9^/L. Prophylaxis for PJP cannot rely solely on CD4^+^ T counts in non-HIV immunocompromised patients. Whether *CMV* infection increases the risk of PJP remains to be further investigated.

## Introduction

*Pneumocystis jirovecii* pneumonia (PJP) is a potentially life-threatening opportunistic infection that predominantly affects immunocompromised hosts including those with *human immunodeficiency virus* (HIV). Other non-HIV immunocompromised hosts including solid organ transplantation, hematopoietic stem cell transplantation, hematologic malignancies, connective tissue diseases, solid tumors and other autoimmune inflammatory disease that need systemic corticosteroids and/or immunosuppressant therapy [1]. In recent years, with the advent of antiretrovirals and prophylaxis with trimethoprim-sulfamethoxazole (TMP-SMX), the incidence of PJP in the HIV patients has decreased significantly. However, as the widespread use of immunosuppressive drugs and antitumor therapy, the broader application of solid organ and bone marrow transplantation, the prevalence of PJP in non-HIV immunocompromised patients has gradually increased [2]. Studies have shown that PJP in non-HIV patients have a more progressive course and higher mortality rate (ranging from 30% to 60%) than HIV patients [3, 4]. Given the serious consequence of PJP in non-HIV immunocompromised patients, it is important to find the risk factors which could be used to predict the occurrence of PJP. Guidelines for preventing PJP in HIV patients are based on CD4^+^ T cells less than 200 cells/µl [5]. There are no consensus guidelines for prophylaxis of PJP in non-HIV immunocompromised patients. Previous study has reported that lymphopenia, corticosteroids and a low count of CD4^+^ T cells are risk factors for the development of PJP in adults with autoimmune inflammatory disorders [6]. However, whether such cut-off value of CD4^+^ T cells < 200 cells/µl can be used as a PJP prophylaxis in non-HIV immunocompromised patients is inconsistent between different academics [7, 8].

Due to the difficulty of culturing* Pneumocystis jirovecii* (*P. jirovecii*) in vitro and the low detection rate of traditional staining microscopy [9], the high prevalence of opportunistic pathogens and mixed infections in immunocompromised patients, there is an urgent need of a diagnostic technique that can rapidly detect a wide range of pathogens. Metagenomic next-generation sequencing (mNGS) is a high-throughput gene sequencing technology that has the advantages of fast, comprehensive, and high sensitivity for the detection of pathogenic microorganisms in lower respiratory tract infection[10], especially in mixed pulmonary infection [11]. Previously, the application of mNGS in the diagnosis of PJP has been reported in several studies [9, 12]. It has showed that mNGS of BALF is more specific and sensitive in the diagnosis of PJP in non-HIV patients than the traditional laboratory test, so mNGS was recommended as a PJP diagnosis technique in immunocompromised patients. The study of Sun et al. [12] showed that non-HIV immunocompromised patients were more likely to manifest a mixed infection of *P. jirovecii* with other pathogens (85.8%). However, the distribution of co-pathogens in non-HIV immunocompromised patients with PJP and non-PJP pneumonia is still unclear.

## METHODS

### Study Design and subjects

**Fig. 1:**
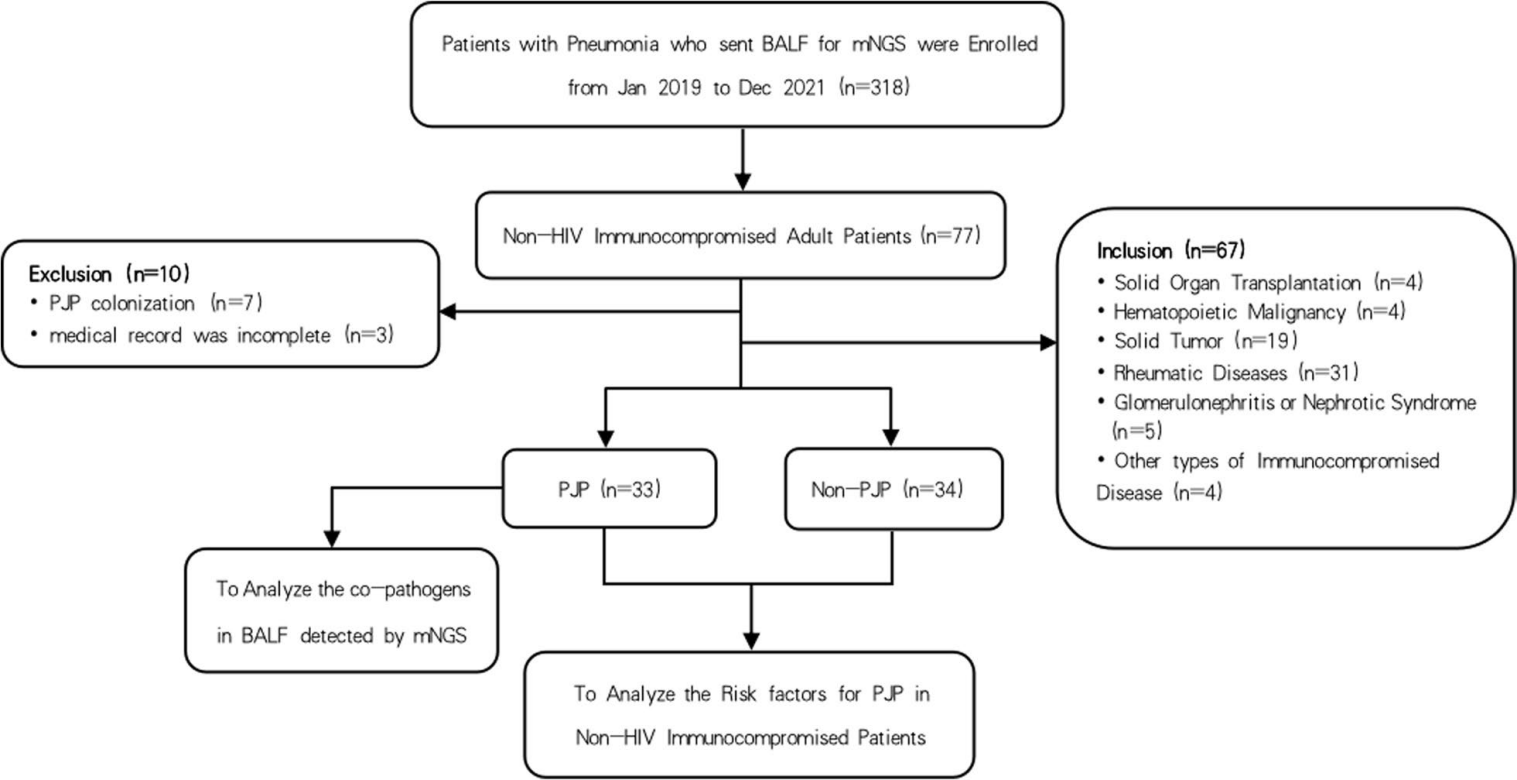
Overview of the study design. *BALF* bronchoalveolar lavage fluid, *PJP* *Pneumocystis jirovecii* pneumonia, *mNGS* metagenomic next-generation sequencing

In this retrospective study, a total of 318 hospitalized patients with pneumonia whose bronchioalveolar lavage fluid (BALF) were collected for mNGS were screened from 1 January  2019 to 31 December 2021 in the Department of Respiratory and Critical Care Medicine of Fujian provincial hospital (Fuzhou, China). 33 non-HIV immunocompromised adult patients with PJP were recruited as study subjects, named as observation group (PJP group). The immunocompromised condition was defined as follows [13]: ① primary immune deficiency diseases; ② solid organ transplantation or hematopoietic stem cell transplantation; ③ hematological malignancies; ④ solid tumors receiving anti-tumor therapy (e.g., chemotherapy), excluding localized skin cancers or early-stage cancers; ⑤ taken glucocorticoids therapy with ≧ 20 mg prednisone or equivalent daily for ≧ 14d or a cumulative dose > 600 mg prednisone; ⑥ taken antirheumatic drugs, biological immune modulators or immunosuppressants (e.g., cyclosporin, cyclophosphamide, methotrexate). PJP diagnosis criteria were as follows: ① clinical symptoms with fever, cough, or shortness of breath; ② Chest computed tomography (CT) showed multiple round-glass interstitial exudation, reticulate or consolidated shadows in both lung; ③ BALF were collected for mNGS and *Pneumocystis jirovecii* was detected. Confirmed diagnosis of PJP was made if items ①–③ were met. Meanwhile, 34 non-HIV immunocompromised patients admitted to our department because of pulmonary infections and confirmed as having non-PJP pneumonia during the same time interval were enrolled as control group (non-PJP group). Patients were excluded if they met any of the following criteria: (1) HIV infection; (2) age < 18 years old; (3) medical record was incomplete. The clinical diagnosis of PJP or non-PJP pneumonia was made by two Senior expert pulmonologists (Yusheng Chen and Nengluan Xu) based on clinical symptoms, laboratory findings, Chest computed tomography, etiological results by mNGS and clinical response to the treatment. The overall study design was shown in Fig 1.

### Sample collection of BALF and etiological diagnosis

Bronchoalveolar lavage was performed by experienced bronchoscopists after local anesthesia with 2% lidocaine in accordance with the standard procedures at Fujian Provincial Hospital. An equal volume of sterile saline was instilled into the affected bronchial segments based on the CT images. BALF was retrieved under negative pressure, put into sterile containers, immediately send for mNGS and conventional methods. BALF was centrifuged to collect the sediment for *Pneumocystis jirovecii* identification under the microscope after Grocott′s methenamine silver (GMS) staining.

### DNA extraction, construction of library and sequencing

The BALF specimens were sent to Hangzhou JieYi Biotechnology Co., Ltd. (Fuzhou) for mNGS. DNA was extracted from BALF samples using NGS master™ Nucleic Acid Extraction Kit (MD053, Hangzhou JieYi). The library was constructed by the metagenomic DNA library building kit (MD001T, Hangzhou JieYi), and purified by purification kit (MD053, Hangzhou JieYi). Library pools were then loaded onto an Illumina Next Seq™ 550Dx next-generation sequencing platform for 75 bp, single-end sequencing to generate approximately 20 million reads for each library. A negative control sample was processed and sequenced in parallel in each sequencing run for quality control.

### Bioinformatics analysis

The microbial sequence reads from mNGS data were analyzed by Gentellix software to get the abundance of each microorganism. High-quality sequencing data were generated by removing low-quality reads, as well as those shorter than 35 bp. Human host sequences were identified and subtracted by mapping to human reference genome (hg38). A set of taxonomic references similarly to the National Center for Biotechnology Information (NCBI) for identification of the microorganisms were built. The database consisted of more than 20,000 pathogens, including viruses, bacteria, fungi, parasite and other pathogens. The microbial reads remaining after the removal of low-complexity reads were identified and classified by alignment to the reference databases. To minimize read-depth variation, the actual read numbers of each identified organism were normalized to reads per million (RPM). The positive detection results for virus were defined as ≥ 3 reads from distinct genomic regions. A positive bacterial or fungal detection was reported if the reads RPM-ratio, which was defined as RPM (sample) /RPM (negative control), was ≥ 5. Meanwhile, bacterial RPM should be ≥ 10 and fungal RPM should be ≥ 2. To provide the most accurate results, workflows for detecting and reporting results have preestablished metrics for quality control by the defined criteria [14].

### Statistical analysis

Continuous variables are presented as medians and interquartile ranges while categorical variables are presented as counts and percentages. Comparative analyses were conducted by using the chi-square test or Fisher’s exact test for categorical variables, and Student’s t-test or Mann–Whitney U test for continuous variables. SPSS 26.0 (IBM Corp., USA) software was used for statistical analysis. P values < 0.05 were considered significant, and all tests were 2-tailed.

## Results

### The baseline data of the participants

The median ages and gender compositions of the two groups were similar. There was no significant difference in the underlying disease and PJP prophylaxis between the two groups. In this study, connective tissue disease accounted for the highest proportion of immunocompromised conditions. Compared to those with non-PJP pneumonia, receiving systemic corticosteroids combined with immunosuppressants was much common in patients with PJP. The baseline data of the participants are summarized in Table 1.

**Table 1:** The basic clinical data of enrolled participants

Characteristics (n [%]or median [IQR])	PJP patients (N = 33)	Non-PJP patients (N = 34)	Statistical value	*P* value
Male	20 (60.61)	24 (70.59)	0.740	0.390
Age (years)	61 (48,67)	61 (50,69)	− 0.217	0.829
*Underlying illnesses*				
Diabetes mellitus	8 (24.24)	7 (20.59)	0.129	0.720
Hypertension	12 (36.36)	12 (35.29)	0.008	0.927
Chronic pulmonary disease	14 (42.42)	7 (20.59)	3.711	0.054
Chronic heart failure	1 (3.03)	1 (2.94)	–	1.000
*Immunocompromised conditions*				
Solid organ transplantation	4 (12.12)	0 (0)	2.490	0.115
Hematologic malignancies	2 (6.06)	2 (5.88)	0.000	1.000
Rheumatic diseases	15 (45.45)	16 (47.06)	0.017	0.895
Solid organ tumors	6 (18.18)	13 (38.24)	3.315	0.069
Glomerulonephritis or nephrotic syndrome	3 (9.09)	2 (5.88)	0.001	0.972
Other types of immunocompromised disease	3 (9.09)	1 (2.94)	0.299	0.585
Use of corticosteroids only	7 (21.21)	3 (9.09)	1.166	0.280
Use of immunosuppressants only	3 (9.09)	3 (9.09)	0.000	1.000
Corticosteroids with immunosuppressants	14 (42.42)	2 (5.88)	12.302	0.000
PJP prophylaxis	1 (3.03)	2 (5.88)	0.000	1.000

*PJP* *Pneumocystis jirovecii* Pneumonia

### The clinical characteristics, laboratory findings and prognosis of PJP and non-PJP patients

Fever and dyspnea were much common symptoms in PJP patients, and lymphocyte counts were significantly lower in the PJP group than in the non-PJP group (*P* = 0.044) (Table 2). The serum levels of (1,3)-beta-d Glucan (BDG) and lactate dehydrogenase (LDH) were significantly higher in the PJP patients than the non-PJP patients (*P* < 0.05). 78.78% of the PJP patients had respiratory failure and 27.27% of the patients died within 30 days. However, there was no statistically significant difference in the rate of respiratory failure and 30-day mortality between the two groups.

**Table 2:** Clinical characteristics, laboratory findings and prognosis of PJP and non-PJP patients

Characteristics (n [%]or median [IQR])	PJP patients (N = 33)	Non-PJP patients (N = 34)	Statistical value	*P* value
Clinical symptoms Cough	21 (63.64)	27 (79.41)	2.051	0.152
Expectoration	15 (45.45)	19 (55.88)	0.729	0.393
Fever	25 (75.76)	14 (41.18)	8.232	0.004
Dyspnea	25 (75.76)	17 (50.00)	4.750	0.029
White blood cells (×10^9^/L)	7.90 (6.20, 10.80)	10.15 (7.68, 12.28)	− 1.825	0.068
Neutrophils (×10^9^/L)	6.92 (5.30, 8.95)	7.85 (5.23, 87.33)	− 0.884	0.377
Lymphocytes (×10^9^/L)	1.00 (0.40, 2.70)	1.50 (0.95, 6.30)	− 2.015	0.044
PCT (ng/mL)	0.42 (0.27, 2.14)	0.24 (0.10, 3.29)	− 1.519	0.129
ESR (mm/h)	66.00 (43.25, 82.75)	63.00 (35.00, 81.50)	− 0.341	0.733
CRP (mg/L)	44.30 (19.05,137.50)	53.20 (16.37, 156.00)	− 0.083	0.934
ALB (g/L)	29.00 (24.00, 34.00)	32.50 (29.00, 35.00)	− 12.571	0.000
LDH (U/L)	461.50 (283.25, 681.25)	196.50 (301.00, 378.00)	4.346	0.000
> 250	28 (84.85)	19 (55.88)	6.710	0.010
Serum BDG (ng/L)	53.3 (7.75, 310.9) (n = 26)	9.00 (5.00, 25.00) (n = 23)	− 2.395	0.017
> 80	12 (46.15)	3 (13.04)	6.299	0.012
> 200	10 (38.46)	0 (0)	8.873	0.003
Lymphocyte subsets (/µl)				
CD3^+^ T cell number	419.00 (213.00, 1014.50)	602.00 (326.00, 887.75)	− 0.934	0.350
CD4^+^ T cell number	195.00 (107.00, 416.50)	321.00 (133.50, 473.25)	− 1.329	0.184
< 300	21 (63.64)	16 (47.06)	1.861	0.172
< 200	17 (51.52)	12 (35.29)	1.795	0.180
CD8^+^ T cell number	176.00 (79.50, 403.00)	235.50 (107.50, 374.00)	− 0.671	0.502
CD4^+^ T cell /CD8^+^ T cell	1.03 (0.55, 2.27)	1.64 (0.71, 2.11)	− 0.596	0.551
NK cell number	30.00 (10.50, 103.75)	72.50 (26.25, 153.75)	− 1.691	0.091
CD19^+^B cell number	55.00 (13.00, 113.00)	113.00 (32.50, 154.75)	− 1.518	0.129
Humoral immunity (g/L)	(n = 22)	(n = 26)		
IgG	8.33 (7.02, 14.85)	12.90 (9.41, 15.68)	− 1.428	0.153
IgA	1.79 (1.29,2.91)	2.16 (1.59, 2.95)	− 1.076	0.282
IgM	0.79 (0.42, 1.51)	0.96 (0.70, 1.51)	− 1.397	0.162
Complement C3	1.07 (1.07, 1.36)	1.16 (0.88, 1.36)	− 1.283	`0.199
Complement C4	0.21 (0.18, 0.27)	0.25 (0.22, 0.33)	− 1.738	0.082
GMS staining (+)	4 (12.12)	0 (0)	2.490	0.115
Admission to ICU	9 (27.27)	15 (44.12)	2.067	0.151
Invasive mechanical ventilation	10 (30.30)	10 (29.41)	0.006	0.936
Severe pneumonia	18 (54.55)	15 (44.12)	0.729	0.393
Respiratory failure	26 (78.79)	21 (61.76)	2.318	0.128
Septic shock	4 (12.12)	5 (14.71)	0.000	1.000
MODS	3 (9.09)	1 (2.94 )	0.299	0.585
30-day mortality	9 (27.27)	9 (26.47)	0.005	0.941

*PJP* *Pneumocystis jirovecii* Pneumonia, *PCT* procalcitonin, *ESR* erythrocyte sedimentation rate, *CRP* C-reactive protein, *ALB* albumin, *LDH* lactate dehydrogenase, *BDG* (1,3)-beta-d Glucan, *GSM* Grocott′s methenamine silver, *ICU* intensive care unit, *MODS* multi-organ dysfunction syndrome

### Logistic multivariate analysis of risk factors for PJP in non-HIV immunocompromised patients

The risk factors of *P* < 0.1 in univariate analysis were included in the binary logistic multivariate analysis. Concomitant use of corticosteroids and immunosuppressants and the absolute peripheral blood lymphocyte counts < 0.7 × 10^9^/L were the risk factors for PJP in non-HIV immunocompromised patients (Table 3).

**Table 3:** Logistic multivariate analysis of risk factors for PJP in non-HIV immunocompromised patients

Risk factors	B	S.E.	Wald	*P *value	*OR* value	95% CI
Corticosteroids with immunosuppressive agents	2.649	0.918	8.335	0.004	14.146	2.341-85
Lymphocytes < 0.7 × 10^9^/L	1.929	0.757	6.495	0.011	6.882	1.561–30.335

*PJP* *Pneumocystis jirovecii* Pneumonia, *OR* odds ratio, *CI* confidence interval

### Mixed infections and co-pathogens detected by mNGS

**Fig. 2:**
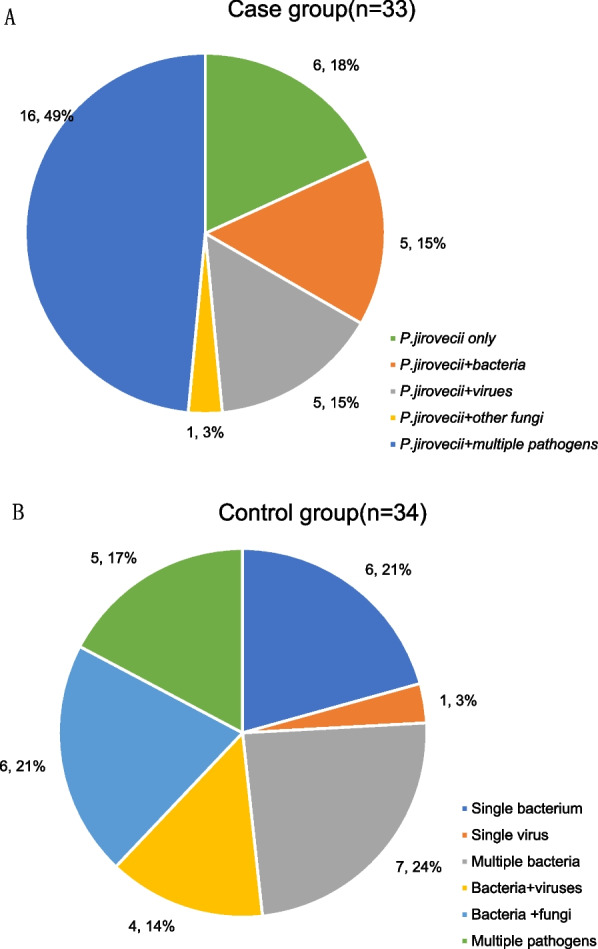
Mixed infections identified by mNGS in case group and control group

**Fig. 3:**
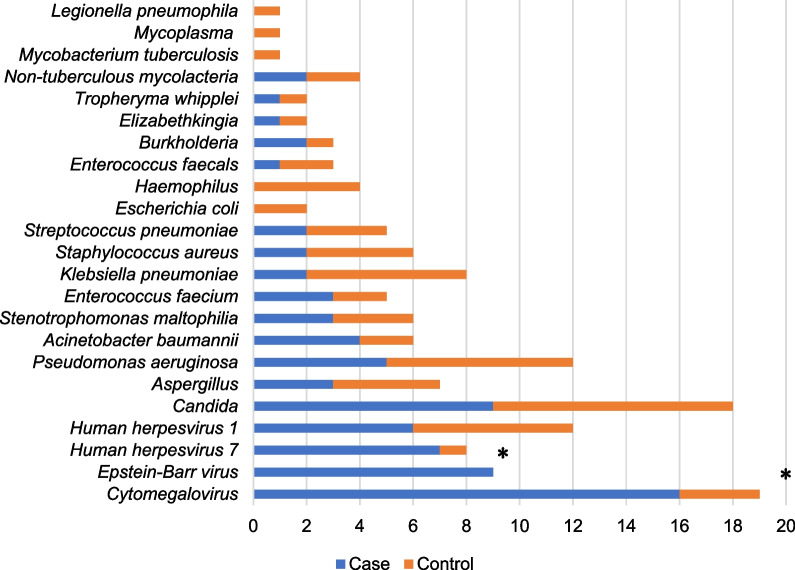
Co-pathogens identified by mNGS in case group and control group. *The case numbers of *Cytomegalovirus* and *Epstein–Barr virus* identified by mNGS in case group were significantly higher than that in control group (*p* < 0.005)

81.82% (27/33) and 64.71% (22/34) mixed infections were identified by mNGS in the PJP group and the non-PJP group separately (Fig. 2). *CMV*, *EBV* and *Candida* were the leading co-pathogens with *P. jirovecii* in the PJP group. The percentages of *CMV* and *EBV* identified by mNGS in the PJP group were significantly higher than those in the non-PJP group [48.48% (16/33) vs. 8.82% (3/34); 27.27% (9/33) vs. 0(0/34)] (*p* < 0.005) (Fig. 3). Other opportunistic pathogens such as *Aspergillus* and *NTM *(*Non-tuberculous mycobacteria*) can also be detected by NGS at the same time.

## Discussion

PJP in non-HIV immunocompromised patients often has no specific clinical symptoms. The serum (1,3)-beta-d Glucan (BDG) test is often used as a non-invasive means to support the diagnosis of PJP. Our study showed that the levels of BDG were much higher in PJP group than in non-PJP group. However, only 46.15% of PJP patients had elevated serum BDG (> 80 ng/L). Morjaria [15] showed that a serum BDG level of > 200 ng/L favors *P. jirovecii* infection over colonization in cancer patients. BDG with cut-off level 200ng/L combined with a positive PCR result of *P. jirovecii* had 92% sensitivity and 90% specificity in the diagnosis of PJP in HIV patients [16]. In this study, we found that only 38.46% PJP patients with a serum BDG level of > 200ng/L. So, BDG > 200ng/L is inadequate for establishing the diagnosis of PJP in non-HIV immunocompromised patients. A meta-analysis [17] showed that the specificity of BDG is inadequate to diagnose PJP and the sensitivity of BDG is insufficient to exclude PJP. In accordance with the findings of Chen [18], we observed that an increase of serum LDH (> 250U/L) was noted in 84.85% of the PJP patients, and the levels of LDH were significantly higher in the PJP patients than in the non-PJP patients. However, LDH was also elevated in 55.88% of the non-PJP patients. As LDH was commonly elevated in many diseases, the performance of using LDH for prediction PJP was limited. Thus, confirmation of the presence of *P. jirovecii* in respiratory secretions remains a key measure for the diagnosis of PJP. As was reported by Chen [19], the positive rate of *P. jirovecii* detected by traditional smear staining method is significantly lower than that of molecular detection method (9.72% vs. 90.63%). In our study, *P. jirovecii* were observed at direct microscopic examination by GMS staining in only 12.12% (4/33) BALF samples of the PJP patients.

Due to the difficulty of effectively identifying PJP based on clinical manifestations and conventional diagnostic techniques, and the high prevalence of serious mixed infection with poor prognosis in immunocompromised patients [20], it needed to develop a diagnostic technique that can detect a wide range of pathogens. Zhan et al. [20] have confirmed that metagenomic next-generation sequencing (mNGS) benefited the pathogen diagnosis and clinical management of pneumonia in immunosuppressed patients. mNGS is a useful diagnostic tool with good performance for the diagnosis of PJP and the detection of co-pathogens [9]. Consistent with the findings of Sun [12], we found that mixed infections were detected in 81.82% of PJP patients and 64.71% of non-PJP patients by mNGS. In our study, we found that *CMV* was the leading co-pathogens in the PJP patients and the detection rate of *CMV* in the PJP patients was significantly higher than that in the non-PJP patients(*p* < 0.005). A meta-analysis [21] showed that *CMV* infection significantly increased the risk of PJP in solid organ transplant recipients. Besides that, other research has found [22] that 30-day mortality was higher in the *CMV* co-infection group as compared to the group with PJP alone. However, our study did not find that *CMV* co-infection was associated with 30-day mortality in the PJP patients. Whether *CMV* infection increases the risk of *P. jirovecii* infection and the relationship between *CMV* and *P. jirovecii* in non-HIV immunocompromised patients remains to be further investigated. In addition, we found that *Pseudomonas aeruginosa*, which has the highest detection rate in the respiratory specimens in our hospital at the same time, ranked first among mixed bacterium in both PJP patients and non-PJP patients. It suggests that we need to refer to the local pathogen distribution when selecting empiric anti-infective therapy for immunocompromised patients.

It was reported by Roux [4], the rate of hospital deaths in patients with PJP was significantly higher for non-AIDS patients versus AIDS patients. A meta-analysis [23] showed that the overall mortality for non-HIV patients with PJP was 30.6%. In our study, we found that the 30-day mortality in the PJP patients was 27.27%. Because of the poor prognosis of PJP in non-HIV immunocompromised patients, identification of specific risk factors for the development of PJP would help guide preventive treatment early. Lymphopenia and low CD4^+^ T cell counts contribute to the risk of PJP in HIV patients, and guidelines for preventing PJP in HIV patients are based on CD4^+^ T count < 200 cells/µl. However, the relationship between lymphocyte counts and the risk of PJP in non-HIV immunocompromised patients is not clear. Lower absolute lymphocyte count at diagnosis date was strongly associated with PJP in solid organ transplantation recipients, and odds of PJP infection were high with lymphocyte count < 0.5 × 10^9^/L [24]. In our study, we found that lymphocyte counts were lower in PJP patients than in non-PJP patients, which was consistent with the findings of Tang et al [25]. It appears that lymphocyte count less than 0.7 × 10^9^/L may be a helpful guidepost in determining PJP prophylaxis in immunocompromised patients. A systematic review [8] showed that CD4^+^ T cell count < 200/µl is a sensitive biomarker to identify non-HIV immunocompromised patients who are at risk of PJP. However, we found that there are 48.48% PJP patients with CD4^+^ T cell count > 200/µl and 35.29% non-PJP patients with CD4^+^ T cell count < 200/µl. The CD4^+^ T cell count < 200/µl was not statistically significant between PJP patients and non-PJP patients. It suggests that unlike patients with HIV, CD4^+^ T cell count < 200/µl cannot be used as a guide for preventive treatment of PJP in non-HIV immunocompromised patients, consistent with the findings of other scholars [7].

Glucocorticoids are currently regarded as the most common predisposing factor for PJP [26]. In this study, 81.82% of the PJP patients used systemic glucocorticoids, of which 60.61% were treated by glucocorticoids combined with immunosuppressants. The study by Rekhtman et al. [27] found that compared with patients who only used immunosuppressants, patients who received hormones combined with immunosuppressants had the highest relative risk of PJP infection. Our study also found that glucocorticoids combined with immunosuppressants was the risk factor for PJP in non-HIV immunocompromised patients. In addition, Rekhtman [27] found that the incidence of PJP infection was significantly higher in patients using hormones and immunosuppressants than the incidence of serious side effects caused by SMZ prophylaxis. The benefits outweigh the risks due to drug side effects [28], suggesting the need for PJP prophylaxis in patients receiving combined immunosuppressant and glucocorticoid therapy. However, the daily dose and duration of glucocorticoid used in patients predisposing to PJP remain unknown. Some report [29] showed that steroid doses are insufficient for PJP risk stratification and identification of prophylactic needs in autoimmune neuromuscular disorders.

This study had several limitations. First, it was a single-center retrospective study with unavoidably intrinsic bias. Immunocompromised conditions include a large group of diseases, each of which is highly heterogeneous. Study results may be inconsistent in different immunocompromised populations. In this study, although a variety of immunocompromised diseases were included, no hematopoietic stem cell transplant patients were included, and the highest proportion of participants was connective tissue disease, which would have biased the results Second, lymphocyte counts were only examined at admission but not before onset of illness in our study, so we cannot conclude causation between exposure to lymphopenia and outcome of PJP acquisition. Future studies analyzing lymphocyte counts preceding PJP diagnosis are required to better characterize risk factors for PJP acquisition in non-HIV immunocompromised patients. Finally, due to the small number of cases in this study, it was not possible to further explore the risk of PJP infection in immunocompromised patients with different doses and courses of glucocorticoid, as well as different types of immunosuppressants. Considering the different patients and sample sizes included in different retrospective studies, the conclusions of risk factors for PJP infection in non-HIV immunocompromised are inconsistent, and a multicenter prospective study is needed. The relationship between co-pathogens, especially *CMV* and *P. jirovecii* in non-HIV immunocompromised patients needed to be further studied.

## Conclusion

There were not specific clinical symptoms of PJP in non-HIV immunocompromised patients and the serum levels of BDG and LDH show inadequate to diagnose PJP. Due to the low positive rate of *P. jirovecii* detected by traditional microscopy method and the high mixed infections in non-HIV immunocompromised patients, mNGS could be recommended as a PJP diagnosis technique in immunocompromised patients. Non-HIV immunocompromised patients who concomitant use of corticosteroids and immunosuppressant with lymphocyte counts < 0.7 × 10^9^/L need to be vigilant about the occurrence of PJP. Unlike HIV patients, prophylaxis for PJP cannot rely solely on CD4^+^ T counts < 200 cells/µl in non-HIV immunocompromised patients. *CMV* was the leading co-pathogens in non-HIV immunocompromised patients with PJP. However, whether *CMV* infection increases the risk of PJP needs to be confirmed by further research.

## Data Availability

The data was obtained from a database under Fujian Provincial Hospital, which are not publicly available due to institutional review board limitation but are available from the corresponding author on reasonable request.

## Abbreviations

PJP: *Pneumocystis jirovecii* Pneumonia
mNGS: Metagenomic next-generation sequencing
BALF: Bronchoalveolar lavage fluid
LYM: Lymphocytes
ALB: Albumin
HIV: *Human immunodeficiency virus*
LDH: Lactate dehydrogenase
BDG: (1,3)-beta-d Glucan
*CMV*: *Cytomegalovirus*
*EBV*: *Epstein–Barr virus*
SMZ: Trimethoprim–sulfamethoxazole
CT: Computed tomography
GSM: Grocott's methenamine silver
NCBI: National Center for Biotechnology Information
RPM: Reads per million
PCT: Procalcitonin
ESR: Erythrocyte sedimentation rate
CRP: C-reactive protein
MODS: Multi-organ dysfunction syndrome
ICU: Intensive care unit
IQR: Interquartile range
OR: Odds ratio
CI: Confidence interval
*NTM*: Non-tuberculous mycobacteria
